# Automatic image quality evaluation in digital radiography using for‐processing and for‐presentation images

**DOI:** 10.1002/acm2.14285

**Published:** 2024-02-05

**Authors:** Ioannis A. Tsalafoutas, Shady AlKhazzam, Virginia Tsapaki, Mohammed Hassan Kharita

**Affiliations:** ^1^ Medical Physics Section OHS Department Hamad Medical Corporation Doha Qatar; ^2^ NAHU ‐ Dosimetry and Medical Radiation Physics Section IAEA Vienna Austria

**Keywords:** digital radiography, image quality, phantoms, post‐processing

## Abstract

**Purpose:**

To investigate the impact of digital image post‐processing algorithms on various image quality (IQ) metrics of radiographic images under different exposure conditions.

**Methods:**

A custom‐made phantom constructed according to the instructions given in the IAEA Human Health Series No.39 publication was used, along with the respective software that automatically calculates various IQ metrics. Images with various exposure parameters were acquired with a digital radiography unit, which for each acquisition produces two images: one for‐processing (raw) and one for‐presentation (clinical). Various examination protocols were used, which incorporate diverse post‐processing algorithms. The IQ metrics’ values (IQ‐scores) obtained were analyzed to investigate the effects of increasing incident air kerma (IAK) on the image receptor, tube potential (kVp), additional filtration, and examination protocol on image quality, and the differences between image type (raw or clinical).

**Results:**

The IQ‐scores were consistent for repeated identical exposures for both raw and clinical images. The effect that changes in exposure parameters and examination protocol had on IQ‐scores were different depending on the IQ metric and image type. The expected positive effect that increasing IAK and decreasing tube potential should have on IQ was clearly exhibited in two IQ metrics only, the signal difference‐to‐noise‐ratio (SDNR) and the detectability index (d’), for both image types. No effect of additional filtration on any of the IQ metrics was detected on images of either type. An interesting finding of the study was that for all different image acquisition selections the d’ scores were larger in raw images, whereas the other IQ metrics were larger in clinical images for most of the cases.

**Conclusions:**

Since IQ‐scores of raw and their respective clinical images may be largely different, the same type of image should be consistently used for monitoring IQ constancy and when results from different X‐ray systems are compared.

## 1 INTRODUCTION

Digital radiography (DR) offers many advantages compared to screen‐film imaging, one of the most important being the post‐processing capability using various software algorithms. Post‐processing allows the original image to be processed in various ways—depending on the anatomical area imaged and the respective diagnostic task—to adjust the latitude (dynamic contrast), contrast and spatial resolution, so that the visibility of various anatomical structures and consequently the diagnostic confidence of the interpreting radiologist are enhanced.^1^ Thus, it is not a surprise that in clinical practice when different examination protocols are used to image the same anatomical structures or different post‐processing protocols are applied onto the same raw image, different image quality (IQ) levels are derived.^2^, ^3^, ^4^, ^5^ This is true both for patient radiographs and for radiographic images of physical phantoms used for IQ evaluation.

The physical phantoms used for IQ evaluation typically contain various structures to assess spatial resolution, low contrast, and dynamic contrast. To quantify IQ, human observers were initially used to score the number of visible structures. However, in view of the problems inherent in visual evaluation (intra‐ and inter‐ observer variability, time consuming process^6^), some phantom manufacturers also offer software for automatic evaluation of IQ, which may determine the number of detectable targets and/or calculate metrics such as modulation transfer function (MTF), signal‐to‐noise ratio (SNR), and signal difference‐to‐noise‐ratio (SDNR), also referred to as contrast‐to‐noise‐ratio (CNR). Phantoms and software for contrast‐detail analysis have also been used to quantify image quality and investigate its correlation with clinical image quality.^7^, ^8^, ^9^, ^10^

However, the correlation of some of the IQ metrics derived using phantoms to the IQ of actual patient's images has been recently disputed.^11^ It has been suggested that IQ is best evaluated using two specific IQ metrics: (1) the MTF for the evaluation of spatial resolution, and (2) the detectability index (d’) for the evaluation of low contrast resolution. The d’ is a parameter calculated using the Non‐Prewhitening Model Observer with Eye Filter (NPWE) to include the human visual transfer function, the normalized noise power spectrum (NNPS) and the MTF, to simulate the human observer performance in clinical interpretation tasks, as for example object detectability of contrast‐detail test objects of certain size and shape.^6^, ^12^

To facilitate the wide and frequent application of IQ testing in DR systems, the IAEA has developed a remote and automated solution using a simple, inexpensive phantom and free software called Automated Tool for Image Analysis (ATIA).^6^ The IAEA methodology has been first tested in a pilot study in small number of hospitals, and initial results suggested that it allows for a complete and automated evaluation of the principal performance characteristics of the imaging chain.^12^ To test the IAEA methodology in wide clinical scenarios and remote settings, the IAEA launched in 2021 a Coordinated Research Project (CRP) entitled “Advanced Tools for Quality and Dosimetry of Digital Imaging in Radiology^13^, ^14^.”

Within the context of the IAEA CRP, the IAEA radiographic phantom/ATIA solution has been tested along with three commercial phantoms and respective software solutions in a single DR system under clinical conditions.**^5^** The main question to be answered was whether these phantoms, related software and IQ metrics are sensitive enough to detect subtle changes in IQ, which may occur because of system malfunction or a change in the system's adjustments, which may change the quality and/or the intensity of the X‐ray beam. To answer this question, different exposure parameters, like tube potential (kVp), incident air‐kerma (IAK) levels, tube additional filtration and post‐processing algorithms have been used to simulate such possible changes. The differences observed between phantoms regarding the impact that the above parameters had on IQ metrics’ values (IQ‐scores), were in some cases unexpected and incomprehensible.^5^ It was thought that failure to detect the expected effect that changes in IAK and kVp have on IQ with some commercial phantoms, and failure to detect any effect of additional filtration on IQ with all phantoms, may be due to post‐processing. It was considered that such effects may be concealed because of the superior effect that post‐processing has on certain image adjustments that affect IQ.

To investigate the effect that post‐processing algorithms have on IQ assessment using the IAEA phantom, a rationale and experimental set‐up similar to that of a previously published study was used.^5^ However, the basic difference of this study from the previous study is that the DR system used in the present study produces and stores automatically two images for each acquisition: one for‐processing (henceforth referred to as **raw**) and one for‐presentation (henceforth referred to as **clinical**), both of which are readily available to the user. Therefore, the impact of exposure factor settings on IQ in images with and without post‐processing can be directly compared. This would clarify whether changes in exposure parameters affect both raw and clinical images adequately to produce a detectable change in the IQ metrics recorded, and whether the use of raw over clinical images is preferable when IQ constancy is monitored with the IAEA phantom.

## 2 MATERIALS AND METHODS

**FIGURE 1:**
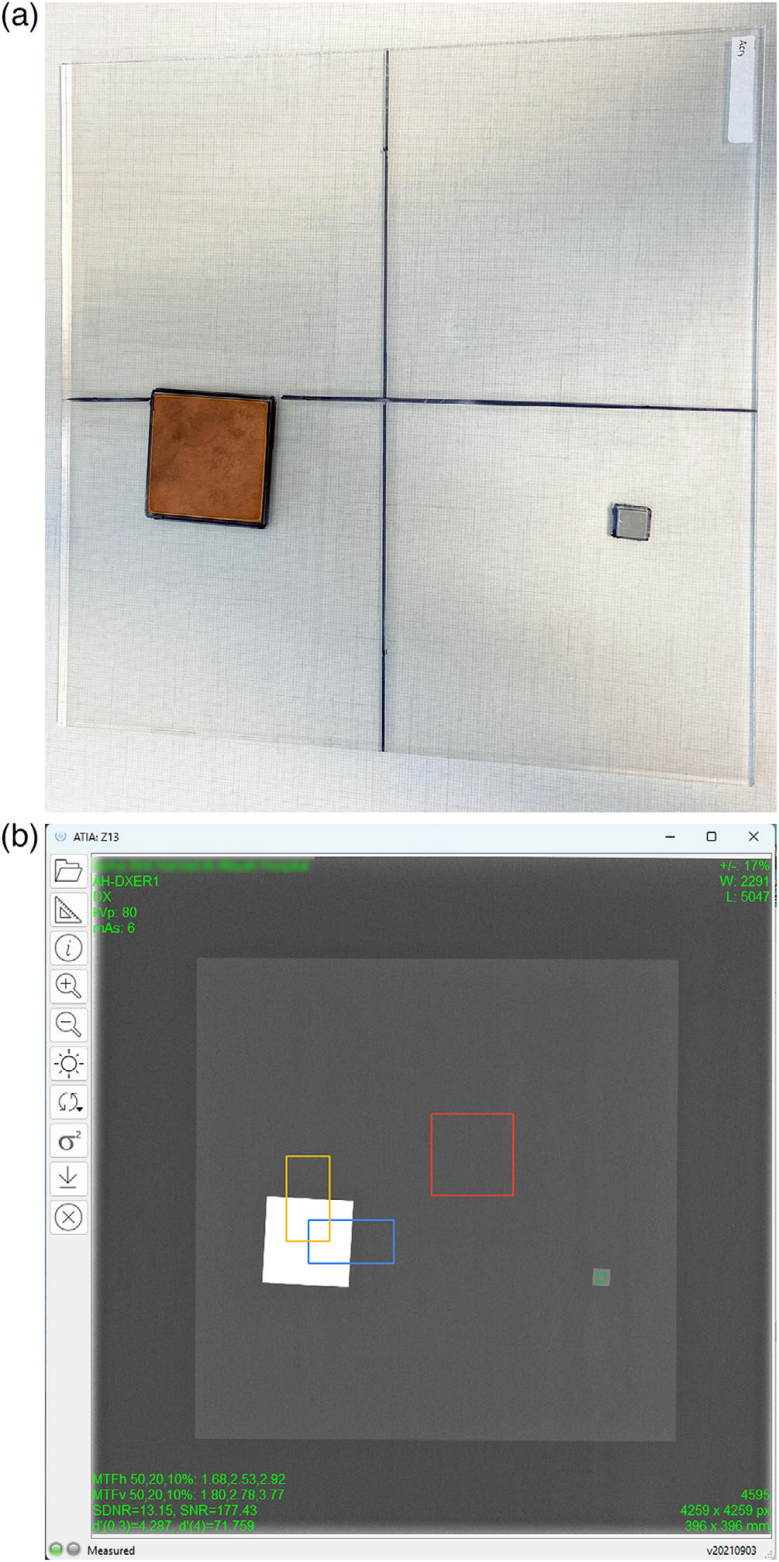
The IAEA radiographic phantom: (a) Photograph, (b) Radiographic appearance of the IAEA phantom within the ATIA software. The regions of interest (ROIs) automatically positioned by the software to calculate the IQ metrics are shown (orange and blue ROIs on two of the Cu square edges, red ROI on the PMMA center and green ROI on the Al square). Note that a 2 mm Cu sheet is also positioned on the X‐ray tube as attenuator.

The IAEA radiographic phantom shown in Figure 1, is made of Poly(methyl methacrylate) (PMMA), Cu and Al according to the IAEA guidance (henceforth referred to as the IAEA phantom). The IQ metrics calculated by the ATIA software are the spatial resolution (MTF @50%, 20%, and 10% in the horizontal and the vertical directions), SNR, SDNR, and the detectability index (d’) for two specific clinical interpretation tasks, which are the detection of 0.3 and 4 mm diameter circular details, both of which are assumed to have an SDNR value equal to that of the 4 mm Al square.**^6^** However, it must be clarified that these circular details are conceptual (their shape is defined by the Fourier transform of disks with diameters D = 0.3 and 4.0 mm) and there are no circular details within the phantom.^12^

The DR unit used to acquire the phantom images was a General Electric (GE) Discovery XR656HD (GE HealthCare, Chicago, Illinois, USA). This unit has been subjected to an extensive quality control (QC) testing prior to the following experiments, and all parameters including tube potential (kVp) accuracy, kVp, incident air kerma (IAK) and Automatic Exposure Control (AEC) system reproducibility, were well within the adopted performance limits.^15^

To investigate the effect of the exposure factors on the IQ, the IAEA phantom was positioned on the radiographic table and the following experiments were performed in one go (without moving the phantom or changing the radiation field size), in which both the raw and clinical images were collected and evaluated using ATIA to automatically calculate the IQ‐scores for all the aforementioned IQ metrics. A free software named DICOM Info Extractor^16^ was used to read the DICOM headers of the phantom images, to derive information about the acquisition and processing parameters of each image.

***Experiment 1***: Five acquisitions were made using the basic acquisition protocol proposed by the IAEA methodology^6^ as follows: Abdomen examination protocol, tube potential of 80 kV, AEC central cell activated (adjusted for an IAK of 2.5 μGy on the image receptor), no additional filtration, and with the antiscatter grid in place. Any variation observed in the IQ‐scores of the IAEA phantom images resulting from these five acquisitions could be attributed to Poisson statistics and/or to minor (≤1%) variations in exposure factors that may occur during repeated exposures under AEC with this radiographic system. The purpose of this experiment was to see how large the IQ‐scores’ differences may be in raw and clinical images, when practically identical exposures and the same examination protocol (i.e., the same post‐processing algorithm) are used. This will serve as a standard of comparison, to decide whether the differences that will be observed in the next experiments should be considered noteworthy or not.

***Experiment 2***: Additional acquisitions were carried out with different examination protocols (Chest PA, Ribs AP, Pelvis AP, Hip AP, Thoracic Spine AP, and Skull LL) which use different preset post‐processing algorithms designed to optimize IQ for the relevant anatomical area. The preset kVp and AEC settings were always switched to 80 kV and central cell activated only (as in the basic acquisition protocol), to test the effect of the post‐processing algorithm alone. The objective of this experiment was to investigate whether IQ‐scores’ changes expected in the clinical images when the examination protocol changes, also occur in the respective raw images.

***Experiment 3***: Acquisitions were made with the basic acquisition protocol, except that IAK on the image receptor was varied manually, using three mAs values below (0.18×, 0.45×, and 0.9×) and three mAs values above (1.4×, 2.2×, and 3.5×) the mAs value selected by the AEC. The objective of this experiment was to investigate the IQ‐scores’ variation with IAK on the image receptor, in both raw and clinical images. As a surrogate of the IAK values on the image receptor, the IAK values at the reference point calculated by the X‐ray system and reported in the DICOM tag named ‘Entrance dose in mGy’ (0040,8302) were used. Though the actual IAK values on the image receptor are much lower (because of the inverse square law, the attenuation by the phantom, the radiographic table and the antiscatter grid), for a constant kVp value the increase or decrease of the entrance dose with respect to the entrance dose value that corresponds to the mAs selection, represents the same increase or decrease respectively, of the IAK value on the image receptor.

***Experiment 4***: Acquisitions were made with the basic acquisition protocol, except that the kVp was varied from 60 to 120 kV in steps of 10 kV, to investigate whether IQ‐scores decrease with increasing kVp, both in raw and clinical images. One acquisition per kVp value was made, except for 80 kV, where two acquisitions were made. The target IAK of the AEC system was kept constant at 2.5 μGy for all kVp settings. Note that 50 kV was not used because too high mAs are required.

***Experiment 5***: Acquisitions were made with the basic acquisition protocol, except that additional filtration of 0.1‐, 0.2‐, and 0.3‐ mm Cu were also used, to investigate whether IQ‐scores’ decrease with the use of harder beam qualities, both in raw and clinical images. Two acquisitions per additional filtration value were made.

## 3 RESULTS

**FIGURE 2:**
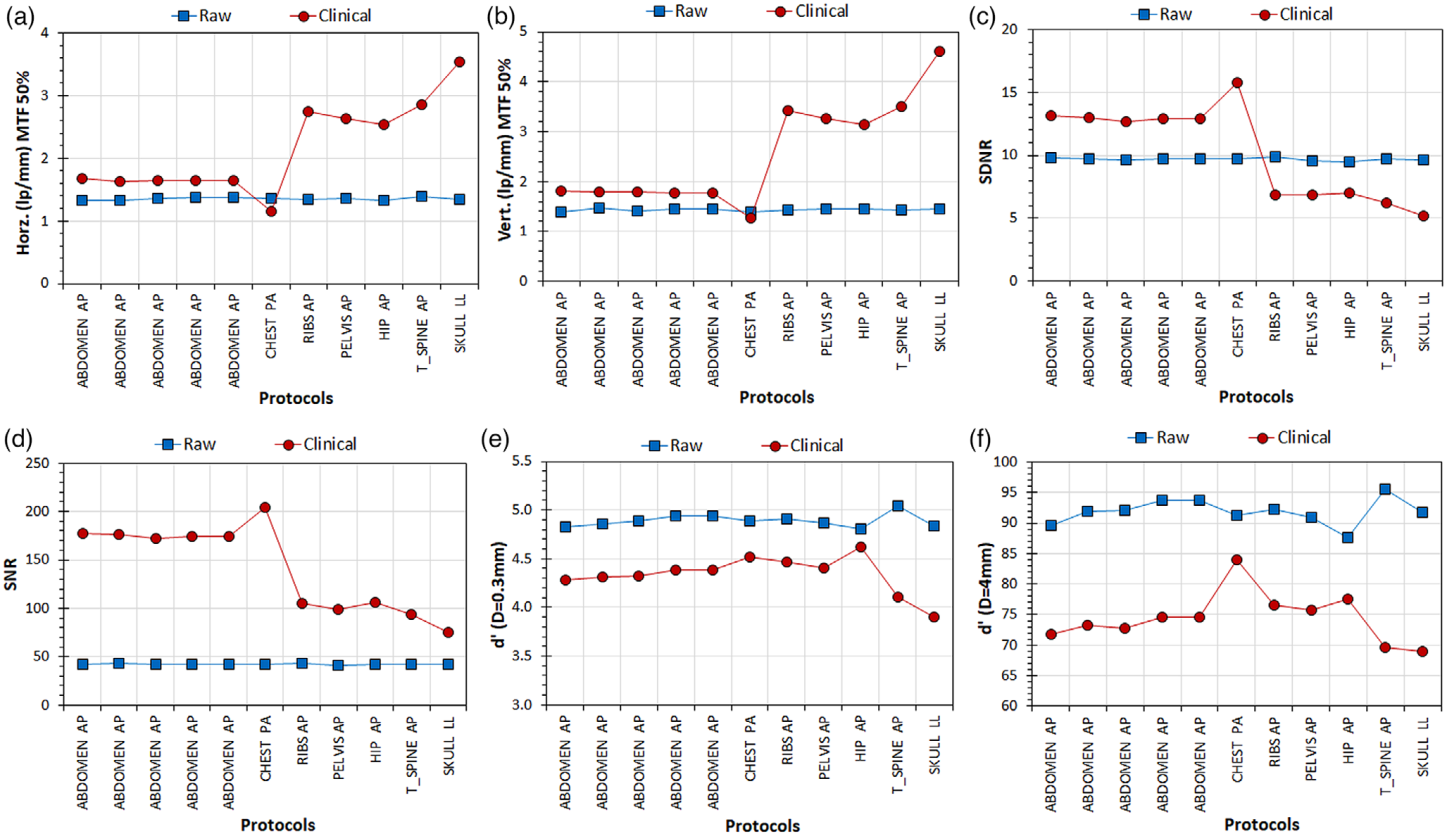
The effect of examination protocols on IQ metrics of the IAEA phantom in raw and clinical images. Repeated acquisitions with the Abdomen AP protocol are also included. (a) Horizontal MTF 50%, (b) Vertical MTF 50%, (c) SDNR, (d) SNR, (e) d’(D = 0.3 mm), (f) d’(D = 4 mm).

**FIGURE 3:**
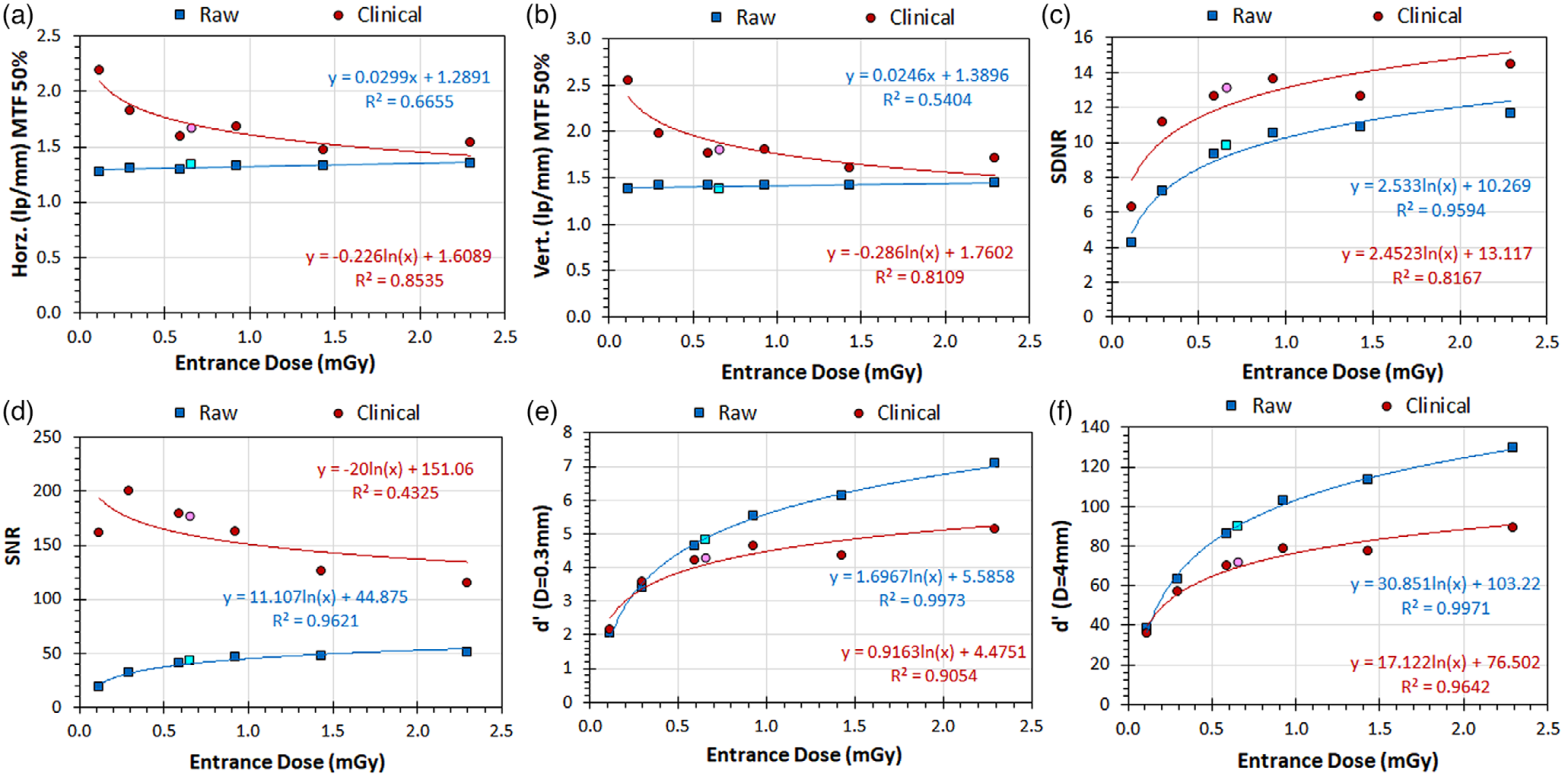
The effect of increasing IAK on the image receptor on IQ metrics of the IAEA phantom, in raw and clinical images. Note that in the X‐axis is shown the Entrance Dose (the IAK calculated by the X‐ray system at the reference point), which is used as surrogate of the IAK on the image receptor. (a) Horizontal MTF 50%, (b) Vertical MTF 50%, (c) SDNR, (d) SNR, (e) d’(D = 0.3 mm), (f) d’(D = 4 mm). The light blue and light pink data points correspond to the raw and the clinical images respectively, acquired using AEC.

**FIGURE 4:**
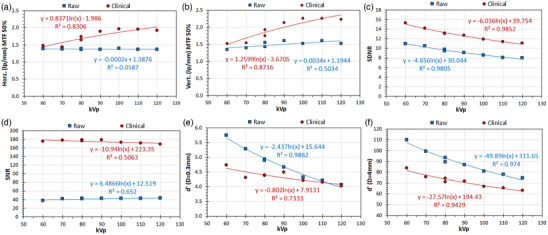
The effect of increasing kV on IQ metrics of the IAEA phantom, in raw and clinical images. (a) Horizontal MTF 50%, (b) Vertical MTF 50%, (c) SDNR, (d) SNR, (e) d’(D = 0.3 mm), (f) d’(D = 4 mm).

The main results of this study are tabulated in Table 1 **(blocks 1−5)**, and selected data are presented in Figures 2, 3, 4.

**TABLE 1:** (Blocks 1−5): Summary of the IQ evaluation results. The max/min ratio is the ratio of the maximum (max) and the minimum (min) values of the IQ metric value observed in each experiment, and it can be used to denote whether there is an effect of the tested parameter on the respective IQ metric (≤1.1: No effect, >1.1 and ≤1.2: Weak, >1.2: Substantial). Trend (correlation) is the *R* ^2^ value of the best fit line used (linear or logarithmic), and the + or—Symbol in front of the *R* ^2^ value denotes a positive or negative trend, respectively. The bigger the *R* ^2^ value the stronger the correlation (*R* ^2^ values <0.3 are denoted with a dash). Regarding the raw versus clinical image IQ‐scores comparisons, with bold are given the mean values of the IQ metrics which are bigger.

Experiment		1. Repeated acquisitions (Poisson statistics)		2. Changing post‐processing algorithm		3. Increasing IAK on image receptor		4. Increasing kVp		5. Increasing filtration	
IQ metrics	Statistical parameter	Raw	Clinical	Raw	Clinical	Raw	Clinical	Raw	Clinical	Raw	Clinical
Horizontal MTF 50% (lp/mm)	min	1.33	1.64	1.32	1.15	1.27	1.47	1.36	1.44	1.31	1.60
	max	1.38	1.68	1.38	3.54	1.35	2.20	1.40	1.96	1.38	1.70
	Mean	1.36	**1.65**	1.36	**2.44**	1.32	**1.71**	1.37	**1.75**	1.35	**1.64**
	max/min	1.04	1.02	1.05	3.07	1.06	1.50	1.03	1.36	1.05	1.06
	trend					+0.67	−0.85	–	+0.83	–	–
Vertical MTF 50% (lp/mm)	Min	1.38	1.76	1.40	1.28	1.38	1.61	1.34	1.52	1.39	1.76
	Max	1.47	1.80	1.45	4.60	1.45	2.56	1.60	2.26	1.47	1.80
	Mean	1.43	**1.78**	1.43	**3.00**	1.41	**1.89**	1.49	**1.95**	1.44	**1.79**
	max/min	1.06	1.02	1.04	3.60	1.05	1.59	1.20	1.48	1.05	1.02
	trend					+0.54	−0.81	+0.50	+0.87	–	–
SDNR	min	9.6	12.7	9.5	5.2	4.2	6.3	8.0	11.1	9.4	12.7
	max	9.8	13.1	9.9	15.8	11.7	14.4	11.0	15.2	9.7	13.4
	Mean	9.7	**12.9**	**9.7**	8.7	9.1	**12.0**	9.3	**12.8**	9.6	**13.0**
	max/min	1.02	1.04	1.04	3.06	2.76	2.29	1.38	1.38	1.03	1.05
	trend					+0.96	+0.82	−0.98	−0.99	–	–
SNR	min	41.9	171.9	41.5	75.1	18.5	114.9	37.1	168.6	41.9	175.8
	max	42.7	177.4	42.9	204.4	51.2	200.6	43.2	178.1	42.8	183.4
	Mean	42.3	**175.1**	42.1	**122.6**	39.8	**160.2**	41.5	**174.5**	42.4	**178.9**
	max/min	1.02	1.03	1.03	2.72	2.76	1.75	1.16	1.06	1.02	1.04
	trend					+0.96	−0.43	+0.65	−0.51	–	–
Detectability Index d’(*D* = 0.3 mm)	min	4.82	4.29	4.80	3.91	2.04	2.16	4.07	4.03	4.69	4.27
	max	4.94	4.38	5.04	4.62	7.08	5.14	5.75	4.73	4.90	4.43
	Mean	**4.89**	4.34	**4.90**	4.34	**4.81**	4.05	**4.77**	4.34	**4.80**	4.35
	max/min	1.02	1.02	1.05	1.18	3.47	2.38	1.41	1.17	1.04	1.04
	trend					+1.0	+0.91	−0.98	−0.73	–	–
Detectability Index d’(D = 4 mm)	min	89.6	71.8	87.6	69.0	38.5	36.1	74.7	63.0	87.9	71.0
	max	93.8	74.6	95.5	84.0	129.6	89.4	110.0	83.7	92.3	74.4
	Mean	**92.2**	73.4	**91.9**	75.3	**89.0**	68.6	**89.0**	71.4	**90.3**	72.9
	max/min	1.05	1.04	1.09	1.22	3.37	2.47	1.47	1.33	1.05	1.05
						+1.0	+0.96	−0.97	−0.94	–	−0.34

***Experiment 1***: Regarding the maximum variation observed in IQ‐scores with repeated identical exposures (Table 1 **‐Block 1**), it was observed that the variation in mAs using AEC was minimal (<1%) when keeping all exposure and geometrical parameters constant. It was thus deduced that any variations that may be observed in IQ‐scores, most likely would be due to Poisson statistics. The maximum variation observed in all IQ‐scores was 6% (ratio = 1.06) for raw and 4% for clinical images. The raw images presented d’ values larger than the clinical images, both for small (D = 0.3 mm) and large (D = 4 mm) circular object diameters. For the rest of the IQ metrics, clinical images outscored raw images.

***Experiment 2***: Regarding the effect of post‐processing algorithm (Table 1 **‐Block 2**), it was observed that the maximum variations observed in IQ‐scores with different examination protocols were much smaller in raw images (largest max/min ratio = 1.09) than the respective maximum variation in IQ‐scores observed in the clinical images (largest max/min ratio = 3.6). d’ was again superior in raw images, though the other IQ metrics were usually superior in clinical images. As can be seen in Figure 2, for the examination protocols where the primary diagnostic task involves bones (the last 5 data points), MTF 50% values are larger compared to those where the primary diagnostic task involves soft tissues or lung parenchyma, but for SDNR and SNR it seems that the opposite is true. For d’ no such correlation was observed.

***Experiment 3***: Regarding the effect of increasing IAK on the IQ‐scores (reported in Table 1 **‐Block 3**), as can be seen in Figure 3, the horizontal MTF 50% exhibited a positive trend (i.e. the MTF 50% increased with increasing IAK), which however numerically was trivial, since the largest max/min ratio was 1.06. The trend was weaker for the vertical MTF 50%. For clinical images the trend was strong for both the horizontal and vertical MTF 50%, but it was negative and numerically considerable (max/min ratios 1.50 and 1.59). This was rather unexpected, considering that for a given post‐processing algorithm, spatial resolution is defined by the pixel size (100 μm) and thus should not be affected by IAK. Regarding SDNR, a strong positive trend (logarithmic) was observed both for raw and clinical images. For SNR, the variation in raw images exhibited a strong positive trend (logarithmic), whereas conversely for clinical images a negative trend (logarithmic) was observed, which if the first data point is ignored it becomes very strong (from −0.43 becomes −0.96). Regarding d’, for both raw and clinical image a very strong, positive trend (logarithmic) was observed with increasing IAK. Therefore, SDNR and d’ follow the same trend with increasing IAK but unlike d’, SDNR is larger in clinical images. In Figure 3e, it can be seen that for the lowest Entrance dose value, d’ is slightly larger for the clinical image. This was the only case where a d’ value was larger for the clinical and not for its respective raw image.

***Experiment 4***: Regarding the effect of increasing kVp on the IQ metrics (Table 1 **‐Block 4**), as can be seen in Figure 4, MTF 50% did not considerably change for raw images, but for clinical images a strong positive trend (logarithmic) was observed. For SDNR, a strong negative trend was exhibited for both raw and clinical images. For SNR, trends and max/min differences were not considerable. On the contrary, strong negative trends (logarithmic) were observed in d’ values, for both raw and clinical images. Therefore, as with increasing IAK, SDNR and d’ follow the same trend with increasing kVp, but unlike d’ SDNR is larger in clinical images.

***Experiment 5***: Finally, regarding the effect of increasing additional filtration on the IQ‐scores (Table 1 **‐Block 5**), it was seen that max/min differences and trends for all metrics are trivial, both for raw and clinical images. Clinical images were superior regarding MTF 50%, SDNR and SNR, but raw images were superior regarding detectability. The fact that d’ does not significantly deteriorate with additional filtrations is in line with the results of the previous study, which however referred to clinical images only.^5^

## 4 DISCUSSION

According to the IAEA methodology, the use of raw images is preferred for IQ evaluation over clinical images.^6^ However, when raw images are not produced by the X‐ray system or are not easy to be accessed (for some X‐ray system manufacturers, access to the service mode is required to access the raw images), the use of the clinical images is a valid option, as long as the same examination protocol (i.e. post‐processing algorithm) used for the first test is consistently used for all subsequent tests.^6^ The preference of raw images seems reasonable considering that the effect of the post‐processing algorithms incorporated in every examination protocol is supposed to be adjusted to optimize the IQ of clinical images depicting human anatomy and may not have the same effect on clinical images of physical phantoms used for IQ evaluation.

When raw images are used, consistency of the IQ‐scores suggests that the calibration of image receptor, X‐ray generator, X‐ray tube and AEC system remains stable. However, with raw images the consistency of the post‐processing protocol effect ‐which is of outmost importance in clinical practice‐ is not checked, something also briefly mentioned in the IAEA publication.**^6^** Therefore, even in systems where raw images are readily available, choosing clinical images instead of raw to monitor IQ, remains a valid alternative. Note that in this case the IAEA methodology suggests as an example the use of the Abdomen examination protocol.^6^

This study verified that with the IAEA phantom the selection of the examination protocol has a strong impact on IQ‐scores of clinical images but does not considerably affect the IQ‐scores of raw images (Figure 2). The IAK increase positively affects d’ and SDNR of both raw and clinical images, but for raw and clinical images SNR follows different trends (Figure 3). On the other hand, the kVp increase (which is preferable from the patient dose perspective), positively affects the MTF of clinical images, negatively the detectability and SDNR of both raw and clinical images but has small impact on SNR (Figure 4). Increasing the additional filtration had no marked effect on IQ‐scores of either raw or clinical images, which is good news considering the significant reduction of patient exposure obtained with increasing additional filtrations and it is in accordance with the results of the previous study regarding clinical images.**^5^**

Overall, MTF 50%, SDNR and SNR of clinical images were larger than the respective values of raw images in most of the experiments, while the opposite was valid for d’. The observation that d’ was larger for raw images than for clinical images (with a single exception shown in Figure 3e), is one of the most interesting findings of this study, since at first it seems contradictory. This is because d’ is supposed to be the metric most closely correlated to clinical IQ, and post‐processing algorithms are applied in clinical images to enhance IQ. A possible answer to this is that post‐processing is intended to optimize the visual detection of the anatomy of interest (commonly found within a complex background) by human observers and not software, though it is supposed that d’ incorporates modeling of human vision. Indeed, d’ is calculated under specific conditions (80 kVp) for a specific clinical interpretation task (detection of conceptual holes of diameters 0.3 and 4 mm of contrast equal to that of the 4 mm Al square in a uniform background of 2 mm Cu and 0.5 mm PMMA), which is not representative of all clinical detection tasks’ conditions. Clinical images of human anatomy contain more than one anatomical structure (targets) with different contrast levels (PV and SDNR differences) from the surrounding structures or tissues (background), and at various brightness (exposure) levels. The processing algorithm considers that all anatomical structures of interest, must be simultaneously visualized by radiologists, even though radiologists may occasionally use manual window width and window level adjustments to better visualize certain parts of the image. However, it has been seen that intense post‐processing aiming to increase the visual contrast may inadvertently misrepresent the actual clinical image.^3^ This may be the reason why some radiography and mammography systems always provide the raw images along with the clinical ones.

In the IAEA methodology document, it is stated that d′ relates subjective measurements such as the SDNR and the MTF, to actual clinical interpretation tasks and through d′, a simple phantom can be directly linked to clinical imaging performance.**^6^** Nevertheless, in view of the above, it remains doubtful whether the IAEA radiographic phantom and the d’ metric, should be used to compare the different post‐processing algorithms that are assigned to different examination protocols, to determine which is superior in real clinical conditions, since the clinical diagnostic tasks may be different for the conditions that each examination protocol and its incorporated post‐processing protocol have been designed for.

Further investigation is required to verify whether raw images of the IAEA phantom present better detectability than the respective clinical images, when using radiography systems from other manufacturers. However, in the context of consistency QC tests, it is imperative that comparisons using d’ values, whether raw or clinical images are used, to be performed always using the same examination protocol. Furthermore, when IQ‐scores obtained in different X‐ray systems using the same IAEA phantom are compared, it should be taken into account that IQ‐scores from raw and clinical images can be largely different, so the comparisons should be limited between images of the same type. The same also applies when it comes to the use of clinical images of different examination protocols.

## 5 CONCLUSION

The study showed that radiographic raw and clinical images present different IQ metrics values which in some cases are radically divergent. Furthermore, their dependence on exposure conditions and post‐processing algorithms do not always follow the same trends. However, d’ was consistently larger in raw images than in the respective clinical images.

Since the detectability index d’ is currently considered to be a more suitable IQ metric than MTF, SNR or SDNR, it is the IQ metric that should be used to answer the question whether image quality improves or deteriorates when exposure factors change, using consistently raw images or the same post‐processing protocol.

## AUTHOR CONTRIBUTIONS

All authors substantially contributed to the conception or design of the research and interpreted results. Data acquisition and initial analysis was made by IAT and SA. All authors contributed to drafting the manuscript or revising it critically for important intellectual content.

## CONFLICT OF INTEREST STATEMENT

The authors have no conflict of interest to state.
